# Net technique for intraocular lens support in aphakia without capsular support

**DOI:** 10.1186/s40942-017-0085-8

**Published:** 2017-08-28

**Authors:** Fernando José De Novelli, Theodomiro Lourenço Garrido Neto, Gabriel de Sena Rabelo, Marcel Eduardo Blumer, Ricardo Suzuki, Ramon Coral Ghanem

**Affiliations:** 1 Sadalla Amin Ghanem Eye Hospital, 35 Camboriu Street, Joinville, SC 89216-222 Brazil; 2 Garrido’s Eye Clinic, Manaus, AM Brazil

**Keywords:** Aphakia, Lens implantation, Intraocular lenses, Postcataract

## Abstract

**Background:**

This paper describes a modified surgical technique for intraocular lens implantation in aphakic eyes with no capsular support.

**Methods:**

Retrospective case series. Seventeen eyes of 17 aphakic patients with no capsule support underwent intraocular lens (IOL) implantation using a standardized technique in which a net was created at the ciliary sulcus plane with two threads forming a net pattern. The net was used as support for the IOL. Follow-up ranged from 6 to 38 months, with an average of 23 months.

**Results:**

In all cases, the IOL was safely implanted and remained stable during the follow-up. In 16 eyes, the IOL remained well centered; in one eye, slight decentration was observed. Distance-corrected visual acuity improved by a mean of 4 lines, from 1.13 (LogMAR) to 0.52 (P = 0.01).

**Conclusion:**

This technique might be especially useful in cases of insufficient capsular support associated with tissue loss or iris atrophy. In these cases, iris fixation is not feasible; thus, the only surgical alternative is IOL scleral fixation.

**Electronic supplementary material:**

The online version of this article (doi:10.1186/s40942-017-0085-8) contains supplementary material, which is available to authorized users.

## Background

Several options are available for the correction of aphakia in cases with absence of support of the posterior crystalline lens capsule. The most conservative nonsurgical options are correction with glasses or contact lenses. Glasses are rarely used because the corrective power required for aphakia results in relative loss of visual acuity, significant anisometropia and unsatisfactory esthetical results. One surgical option is intraocular lens implantation in the sclera through sutures or stabilization in a scleral groove [1–5]. Other options include iris-suture fixated lenses or iris fixation by enclavation and angle-supported anterior chamber phakic intraocular lenses [6, 7].

In this retrospective study, we describe a modified surgical technique that enables the implantation of an intraocular lens in the posterior chamber through a reproducible, relatively low-cost suture that provides safe support for the IOL, as well as good centralization and stability.

## Patients and methods

Following approval by an institutional review board, this retrospective cohort study was carried out from April 2014 to April 2016, according to the principles of the Declaration of Helsinki. All study participants provided written informed consent.

The study was carried out at the Sadalla Amin Ghanem Eye Hospital, and Garrido’s Eye Clinic, operations were conducted from April 2014 to April 2016. Seventeen patients underwent operation and were followed-up for at least 6 months. All surgeries were carried out by two surgeons (FJN and TLG).

Post-op follow-up ranged from 6 to 38 months, with an average of 23 months. A full ophthalmologic examination was performed at all consultations, including best-corrected visual acuity, biomicroscopy, fundoscopy and intraocular pressure measurement.

Posterior pars plana vitrectomy (PPV) was performed due to cases of retinal detachment or displacement of the lens into the vitreous cavity (4 patients). In such cases, vitrectomy should be performed regardless of the type of IOL implantation.

## Surgical technique

Topical 1% mydriacyl was applied twice 15 min before peribulbar anesthesia. Standard asepsis was achieved with periocular 10% povidone-iodine solution and 5% povidone-iodine instillation.

**Fig. 1:**
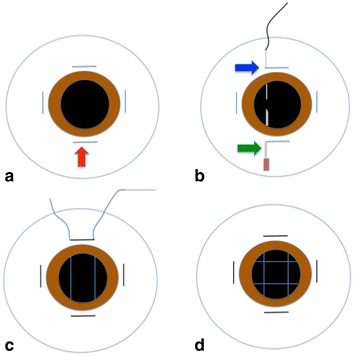
**a** Peritomy in the 4 quadrants and creation of an initial 5-mm-long groove 2.0 mm from the limbus (*red arrow*). **b** A 16-mm needle with suture thread is inserted into the guide needle and later protrudes through the initial sulcus on the opposite side. Therefore, the thread runs through the globe (*blue* to *green arrow*). **c** This procedure is repeated with the same technique and needle but in the opposite direction. A knot is secured and inserted in the initial groove. **d** The same procedure is performed in the perpendicular meridian so that the threads form an efficient support in a netting-shaped structure where the IOL may be safely placed and positioned

A peritomy was performed in the 4 quadrants, and an initial 5-mm-long groove was created 2.0 mm from the limbus (Fig. 1a).

One straight 16-mm needle of a doubled-armed 9-0 polypropylene suture (Ethicon, Inc.) was inserted into the anterior chamber. A 25-gauge needle was inserted 180° away in the contralateral groove to serve as a docking guide. The needle was passed through the guide, exiting on the opposite side (Fig. 1b). This procedure was repeated with the same technique and needle 180° away. The standard distance between the 2 parallel threads was 4 mm. The knot was secured in the initial groove, thus protected by the sclera and preventing conjunctival erosion (Fig. 1c).

The threads formed an efficient support in a netting-shaped structure in which the IOL may be placed and positioned according to the surgeon’s discretion (Fig. 1d).

**Fig. 2:**
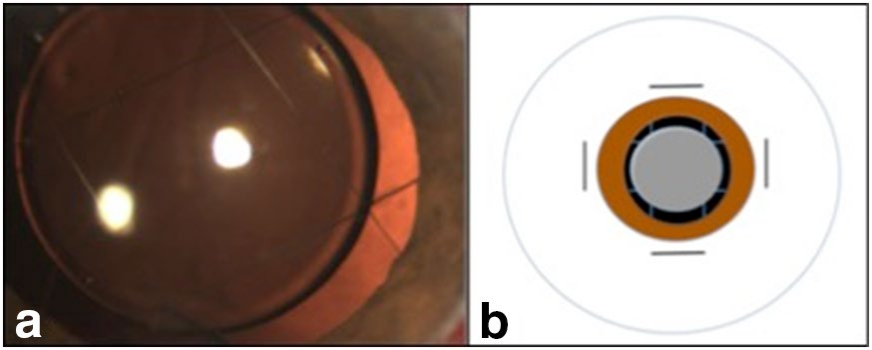
The IOL is inserted underneath the netting, and it remains centered in the right plane without tilting. **a** Image from case report 2. **b** Schematic diagram

The IOL was inserted on the top of the net. Centration was easy, and no tilt was observed (Fig. 2). A 3-piece foldable IOL was implanted with better stabilization. In some cases, repositioning of a previously implanted IOL (single-piece foldable) was performed.

Additional file 1 shows the surgical technique.

## Results

Seventeen eyes of seventeen patients (9 males and 8 females) were enrolled in the study. The mean age was 67 years (range 33–86 years). Preoperative diagnoses included retinal detachment in 1 patient, surgical aphakia in 6 patients, trauma in 4 patients, posterior PPV due to diabetic retinopathy in 1 patient, lens subluxation in 3 patients and IOL explant due to endophthalmitis in 2 patients (Table 1). Follow-up ranged from 6 to 38 months, with an average of 23 months.

**Table 1:** Summary of Patient Data

Patient	Cause	Age	Sex	Follow-up (months)	Visual acuity		FO (postoperative)	BIO
					Preoperative	Postoperative		
1	RD	59	M	9	20/400	20/200	Retina on	IOL centered
2	Surgical aphakia	80	F	12	20/40	20/25	Normal	Slightly dislocated IOL
3	Trauma	34	M	6	CF 1 m	20/80	Retina on	IOL centered
4	PPV (diabetic)	70	F	12	HM	CF 1 m	Retina on with mobilization MP	IOL centered
5	Trauma	33	M	12	CF 1 m	CF 1 m	Retina on with mobilization MP	IOL centered
6	Trauma	56	M	12	LP	20/80	ERM with pseudo-hole	IOL centered
7	Lens subluxation	67	M	8	CF 1 m	CF 1.5 m	PDR	IOL centered
8	Surgical aphakia	80	M	36	20/30 (+1200 SD)	20/30	Normal	IOL centered
9	Lens subluxation (PEX syndrome)	86	F	30	20/100	20/25	Normal	IOL centered
10	Surgical aphakia	68	M	32	20/20 (+1400 SD)	20/20	Normal	IOL centered
11	Endophtalmitis (IOL explant)	80	M	38	LP	20/20	ERM	IOL centered
12	Trauma (IOL dislocation)	83	M	36	20/30	20/25	Normal	IOL centered
13	Lens subluxation (PEX syndrome)	84	F	28	20/25 20/25	Normal	IOL centered	
14	Surgical aphakia	66	F	33	20/25 (+1325 SD)	20/20	Normal	IOL centered
15	Endophtalmitis (IOL explant)	56	F	27	20/400	20/60	Recurrent CME	IOL centered
16	Surgical aphakia	67	F	32	20/80	20/20	Normal	IOL centered
17	Surgical aphakia	72	F	24	20/40 (+1300 SD)	20/30	Chorioretinitis scar juxtafoveal	IOL centered

*FO* fundoscopic, *BIO* biomicroscopy, *RD* retinal detachment, *CF* counting fingers, *HM* hand motion, *LP* light perception, *PPV* pars plana vitrectomy, *MP* macular pigment, *ERM* epiretinal membrane, *PDR* proliferative diabetic retinophaty, *SD* spherical diopters, *PEX* pseudoexfoliation, *CME* cystoid macular edema, *m* meters

Postoperative best-corrected visual acuity (BCVA) was counting fingers in 3 patients (18%), 20/200 in 1 patient (6%), 20/80 in 2 patients (12%), 20/60 in 1 patient (6%), 20/30 in 2 patients (12%), 20/25 in 4 patients (23%), and 20/20 in 4 patients (23%) (Table 1). In all cases, the IOL was safely implanted. No intraoperative complications were observed. After a mean follow-up of 23 months, the lens remained well centered in 95% of cases. Slight dislocation occurred in 1 eye without the need for surgical repositioning. One case presented with pigmented dispersion. No increased pressure, glaucoma or uveitis was observed.

Distance-corrected visual acuity improved by a mean of 4 lines, from 20/200 (1.1 LogMAR) to 20/70 (0.54 LogMAR) (P = 0.01) (we used the LogMAR table from The Journal of Cataract and Refractive Surgery).

## Case report

### Case 1

A 34-year-old male (Patient 3, Table 1) was seen on July 1st, 2014 after left eye trauma caused by a screwdriver. The patient underwent a corneo-scleral suture and hyphema wash for 5 days, which were performed at another health service. Upon examination, the patient presented with hand motion vision, cataract, phacodonesis, and retinal and choroidal detachment diagnosed by ultrasound.

The patient underwent phacoemulsification, IOL implantation in the ciliary sulcus, posterior vitrectomy and silicone oil implantation. The patient progressed to 20/400 vision and presented with an applied retina and a nasally dislocated IOL, which was supported on the ciliary sulcus, with a temporal haptic under the iris.

After 15 months, silicone oil removal was carried out with IOL repositioning according to the netting-shaped structure technique described above, which enabled positioning of the temporal haptic in the ciliary sulcus and IOL centralization.

Visual acuity increased to 20/80; 6 months post-op, the IOL remained well positioned and well centered.

### Case 2

A 56-year-old male patient (Patient 6, Table 1) presented with 5-day-old trauma in the right eye due to splitting wood with an ax. The patient reported loss of vision and pain immediately after the trauma. At our service, the patient presented with increased intraocular pressure, 2 mm hyphema, +/+++ corneal edema, cataract, phacodonesis and visual acuity with light perception. The patient was treated with anti-inflammatory and anti-hypertensive medications. After complete absorption of the hyphema and absence of intraocular inflammation, removal of the crystalline lens was performed.

With an estimated 270° zonular rupture, cataract removal was difficult, with dislocation of a crystalline lens fragment to the vitreous. Pars plana vitrectomy was performed. We chose to perform the netting-shaped structure technique described above for IOL implantation.

The patient progressed to 20/80 visual acuity with a well-positioned lens (Fig. 2a). Vision failed to fully recover due to the formation of an epiretinal membrane and optic disc color change, possibly resulting from trauma.

## Discussion

There are several options for the management of aphakia in the absence of capsular support. Among the definitive surgical options, lens implantation in the anterior or posterior segment is a potential option.

Among lenses implanted in the anterior chamber, the options include iris-suture-fixated lenses or iris fixation by enclavation [6, 8] and angle-*supported* anterior chamber *phakic* intraocular lenses [7], which are rarely used due to complications, such as endothelial loss, pupil distortion and secondary glaucoma [9].

In 1980, Van der Pol and Worst [10] described iris-fixated IOLs for the correction of aphakia in congenital cataract. Compared with earlier designed lenses, the Artisan lens, an iris-fixated polymethyl methacrylate IOL, has been considerably improved and is currently used in many countries for the correction of aphakia and high myopia in patients without capsular support. However, the Artisan lens must be iris-fixated.

Scleral fixation, whether by suture or enclavation through the ciliary sulcus, offers the advantage of maintaining a clear anterior chamber without interfering with the anatomy of the region. However, the surgical technique for this approach is more complex. Transscleral lens fixation may be performed with haptic suturing through the ciliary sulcus or pars plana [1, 3, 5] or without suturing [11–14].

In 2013, Samuel Masket described a technique that involves the creation of safety netting with a double-armed 10-0 polypropylene suture at the ciliary sulcus, which would act as support for handling IOLs lacking adequate capsular support for post-vitrectomy eyes and repositioning of IOLs. He also used this technique for secondary IOL implantation with intrascleral fixation. In this case, the netting acted as scaffolding for IOL implantation. In both situations, the polypropylene netting was removed after IOL fixation or repositioning [15]. The creation of netting at the ciliary sulcus had been previously published in papers reporting silicone oil retention sutures performed to prevent silicone oil migration to the anterior chamber in aphakic eyes [16, 17].

The technique in our study is based on secondary IOL implantation at the ciliary sulcus with the lens supported by a 9.0 polypropylene suture without additional sutures or haptic fixation. Our netting provides full support for both the IOL body and the haptics. Follow-up revealed that the lenses implanted with our technique remained steady and centralized in nearly all cases. This netting provides several support points for the IOL; in addition, since the netting is created 2.0 mm away from the limbus, tilting, a frequent cause of complications in transscleral fixation [18], does not occur.

Another major advantage of this technique is IOL fixation even when iris fixation is not feasible, either due to iris tissue loss or atrophy. This technique could be useful in cases of absence of capsular support in patients with thin sclera; in these cases, scleral fixation is very technically challenging. One disadvantage of using a polypropylene suture for IOL support is that this suture may degrade [19], mainly when it is subjected to ultraviolet rays. An alternative for lessening this complication is the use of Gore-Tex^®^ suture threads; this monofilament material is non-absorbable and has recently shown good results for IOL scleral fixation [20]. Table 2 compares the results of our study with the results of other studies in the literature [21–25].

**Table 2:** Comparing the results of different studies in the literature

Study	Intraocular lens type	Mean follow-up (months)	Eyes (n)	Mean preoperative BCVA	Mean postoperative BCVA	Most frequently complications (%)
Chen et al. [21]	Iris-fixated IOL (Artisan)	36	72	1.18 ± 0.30 LogMAR	0.28 ± 0.18 LogMAR	Pigment precipitates (5.6%); glare and halos (16.7%)
Kwong et al. [22]	SFIOL	33	36	1.31 ± 0.44 LogMAR	0.48 ± 0.38 LogMAR	IOP > 21 mmHg (68.8%); transient corneal edema (42.9%); viteous hemorrhage
	ACIOL	33	46	1.30 ± 0.43 LogMAR	0.32 ± 0.31 LogMAR	IOP > 21 mmHg (100%); transient corneal edema (50%); viteous hemorrhage (10%); hyphema (10%)
Rey et al. [23]	SFIOL	23	38	0.60 LogMAR	0.30 LogMAR	OH (23%), hypotony (15%); CME (8%)
	ACIOL	23	25	0.80 LogMAR	0.30 LogMAR	OH (8%), hypotony (24%); CME (20%)
Agarwal et al. [24]	Fibrin glue assisted sutureless PC IOL	1.5	10	0.30 LogMAR	0.30 LogMAR	None
Kim et al. [25]	SFIOL	16	44	0.60 LogMAR	0.18 LogMAR	IOL dislocation (13%)
	Iris-fixated PC IOL	16	35	0.60 LogMAR	0.28 LogMAR	IOL dislocation (17%); CME (3%)
Net technique		23	17	1.10 LogMAR	0.54 LogMAR	Slightly IOL dislocation (5%); pigment dispersion (5%)

*BCVA* best-corrected visual acuity, *IOL* intraocular lens, *SFIOL* scleral-fixated intraocular lens, *ACIOL* anterior chamber intraocular lens, *IOP* intraocular pressure, *OH* ocular hypertension, *CME* cystoid macular edema, *PC IOL* posterior chamber intraocular lens, *LogMAR* logarithm of the minimum angle of resolution

## Conclusion

The intraocular lens implantation technique in which an IOL is placed anterior to the netting is a relatively low-cost and reproducible option. This technique might be especially useful in cases of insufficient capsular support associated with tissue loss or iris atrophy. In these cases, iris fixation is not feasible; thus, the only correction alternative is scleral fixation of intraocular lenses. The findings of this study demonstrate that the net technique for IOL support is a reproducible, safe and effective option for surgical treatment of aphakia with no capsular support.

The limitations of our study include the small sample size, short follow-up period and absence of a control group. Further comparative studies and longer follow-up periods are essential to assess the safety and effectiveness of this technique.

## Additional files

**Additional file 1.**The video shows the steps of the surgery as described in the paper.

**Additional file 2.** Visual Acuity Conversion Chart
