# Supplementary material for: Net technique for intraocular lens support in aphakia without capsular support
Source: Int J Retina Vitreous. 2017 Aug 28;3:32. doi: 10.1186/s40942-017-0085-8 (PMC5572154; doi:10.1186/s40942-017-0085-8)
Supplement: Supplementary file 2 — Additional file 2. Visual Acuity Conversion Chart [file 40942_2017_85_MOESM2_ESM.pdf]

Visual Acuity Conversion Chart

Line Number	Visual Angle (min)	Spatial Frequency (Cyc/deg)	LogMAR	Distance: % Central Visual Efficiency	Distance: Snellen Equivalent Feet 20/	Distance: Snellen Equivalent Meter 6/	Distance: Decimal	Near: % Central Visual Efficiency	Near: Inches (14/)	Near: Centimeters (35/)	Near: Revised Jaeger Standard	Near: American Point-Type	Near: "M" Notation
−3	0.50	60.00	−0.30	100	10	3.0	2.00	100	7.0	17.5	–	–	0.20
−2	0.63	48.00	−0.20	100	12.5	3.8	1.60	100	8.8	21.9	–	–	0.25
−1	0.80	37.50	−0.10	100	16	4.8	1.25	100	11.2	28.0	–	–	0.32
0	1.00	30.00	0.00	100	20	6.0	1.00	100	14.0	35.0	1	3	0.40
1	1.25	24.00	0.10	95	25	7.5	0.80	100	17.5	43.8	2	4	0.50
–	1.50	20.00	0.18	91	30	9.0	0.67	95	21.0	52.5	3	5	0.60
2	1.60	18.75	0.20	90	32	9.6	0.63	94	22.4	56.0	4	6	0.64
3	2.00	15.00	0.30	85	40	12.0	0.50	90	28.0	70.0	5	7	0.80
4	2.50	12.00	0.40	75	50	15.0	0.40	50	35.0	87.5	6	8	1.0
–	3.00	10.00	0.48	67	60	18.0	0.33	42	42.0	105.0	7	9	1.2
5	3.15	9.52	0.50	65	63	18.9	0.32	40	44.1	110.3	8	10	1.3
–	3.50	8.57	0.54	63	70	21.0	0.29	32	49.0	122.5	–	–	1.4
6	4.00	7.50	0.60	60	80	24.0	0.25	20	56.0	140.0	9	11	1.6
7	5.00	6.00	0.70	50	100	30.0	0.20	15	70.0	175.0	10	12	2.0
–	5.70	5.26	0.76	44	114	34.2	0.18	12	79.8	199.5	11	13	2.3
8	6.25	4.80	0.80	40	125	37.5	0.16	10	87.5	218.8	12	14	2.5
–	7.50	4.00	0.88	32	150	45.0	0.13	6	105.0	262.5	–	–	3.0
9	8.00	3.75	0.90	30	160	48.0	0.13	5	112.0	280.0	13	21	3.2
10	10.00	3.00	1.00	20	200	60.0	0.10	2	140.0	350.0	14	23	4.0
11	12.50	2.40	1.10	17	250	75.0	0.08	0	175.0	437.5	–	–	5.0
–	15.00	2.00	1.18	16	300	90.0	0.07	0	210.0	525.0	–	–	6.0
12	16.00	1.88	1.20	15	320	96.0	0.06	0	224.0	560.0	–	–	6.4
13	20.00	1.50	1.30	10	400	120.0	0.05	0	280.0	700.0	–	–	8.0
16	40.00	0.75	1.60	5	800	240.0	0.03	0	560.0	1400.0	–	–	16.0
20	100.00	0.30	2.00	0	2000*	600.0	0.01	0	1400.0	3500.0	–	–	40.0
30	1000.00	0.03	3.00	0	20000†	6000.0	0.001	0	14000.0	35000.0	–	–	400.0

Bold values are standard logMAR progression.

LogMAR = logarithm of the minimum angle of resolution.

*20/2000 is equivalent to counting fingers @ 2 feet.

†20/20000 is equivalent to hand motion @ 2 feet.

The Journal of Cataract & Refractive Surgery is published monthly by Elsevier Inc., 360 Park Avenue South, New York, NY 10010-1710, USA. Members of the American Society of Cataract and Refractive Surgery (ASCRS) and the European Society of Cataract and Refractive Surgeons (ESCRS) receive the journal as part of their annual dues.

(For Post Office use only: Volume 31 issue 7 of 12)

The subscription rates within North America are as follows: $161.00 (individuals) and $307.00 (institutions). The single-copy price is $36.00.

Subscriptions, inquiries, claims, and changes of address should be sent to Elsevier, Subscription Customer Service, 6277 Sea Harbor Dr., Orlando, FL 32887-4800. Telephone: Toll free (for customers inside U.S./Canada): 800-654-2452. For customers outside the U.S./Canada: 407/345-4000. Fax: 407-363-9661. E-mail: elspcs@elsevier.com.

Members of ASCRS should send change of address notification to Journal of Cataract & Refractive Surgery, 4000 Legato Road, #850, Fairfax, VA 22033, USA; fax (703) 591-0614. Members of ESCRS, to ESCRS, Temple House, Temple Road, Blackrock, Co. Dublin, Ireland; fax (353-1) 209-1112.

No responsibility is assumed by the Journal of Cataract & Refractive Surgery or by Elsevier Inc. for any injury and/or damage to persons or property whether such liability, if any, arises as a matter of product liability, negligence or otherwise, or from any use or operation of any methods, products, instructions, or ideas contained in the material herein. Because of rapid advances in the medical sciences, the publisher recommends that independent verification of diagnosis and drug dosages be made.

Copyright 2005 American Society of Cataract and Refractive Surgery and European Society of Cataract and Refractive Surgeons. All rights reserved. Printed in the USA. None of the contents may be reproduced, stored in a retrieval system (currently available or developed in the future), or transmitted in any form or by any means (electronic, mechanical, photocopying, recording, or otherwise) without prior written permission. All inquiries regarding copyright material from this publication should be directed to Elsevier Ltd., P.O. Box 800, Oxford OX5 1DX, UK; telephone (44-1865) 843830; fax (44-1865) 853333; e-mail, permissions@elsevier.co.uk.

Photocopying policy: In the USA, users may clear permissions and make payments through the Copyright Clearance Center, Inc., 222 Rosewood Drive, Danvers, MA 01923, USA; telephone: (978) 750-8400, fax (978) 750-4744, and in the UK through the Copyright Licensing Agency Rapid Clearance Service (CLARCS), 90 Tottenham Court Road, London W1P 0LP, UK; telephone: (+44) 171-436-5931; fax (+44) 171-436-3986. Other countries may have a local reprographic rights agency for payments.

Advertising (display and classified) inquiries should be addressed to Tino DeCarlo, Elsevier Inc., 360 Park Avenue South, New York, NY 10010, USA; telephone (212) 633-3815; fax (212) 633-3820; e-mail t.carlo@elsevier.com. Commerical reprint inquires should be addressed to Derrick Imasa, Elsevier Inc., 360 Park Avenue South, New York, NY 10010-1710, USA; telephone, (212) 633-3874; fax (212) 633-3820; e-mail, d.imasa@elsevier.com.

Periodicals postage paid at New York, NY, and additional mailing offices. POSTMASTER: Send address changes to Journal of Cataract & Refractive Surgery, 4000 Legato Road, #850, Fairfax, VA 22033, USA.

∞™ This journal is printed on acid-free paper that meets the minimum requirements of ANSI Standard 239.48 (Permanence of Paper).

The Journal of Cataract & Refractive Surgery is cited in Current Contents, Index Medicus, Science Citation Index, and EMBASE.
